# Evaluation of Social Media Utilization by Latino Adolescents: Implications for Mobile Health Interventions

**DOI:** 10.2196/mhealth.4374

**Published:** 2015-09-29

**Authors:** Megan Landry, Amita Vyas, Monique Turner, Sara Glick, Susan Wood

**Affiliations:** ^1^ Department of Prevention and Community Health Milken Institute School of Public Health George Washington University Washington, DC United States; ^2^ Division of Allergy and Infectious Diseases School of Medicine University of Washington Seattle, WA United States; ^3^ Department of Health Policy Milken Institute School of Public Health George Washington University Washington, DC United States

**Keywords:** acculturation, Latino/a, sex, short message service, social networking

## Abstract

**Background:**

Trends in social media use, including sending/receiving short message service (SMS) and social networking, are constantly changing, yet little is known about adolescent’s utilization and behaviors. This longitudinal study examines social media utilization among Latino youths, and differences by sex and acculturation.

**Objectives:**

The purpose of this study was to examine Latino adolescents’ social media utilization and behavior over a 16-month period, and to assess whether changes in use differed by sex and acculturation.

**Methods:**

This study included 555 Latino youths aged 13-19 who completed baseline and 16-month follow-up surveys. Prevalence of social media utilization and frequency, by sex and acculturation categories, was examined using generalized estimating equations.

**Results:**

Women are more likely to use SMS, but men are significantly more likely to SMS a girl/boyfriend (*P*=.03). The use of Internet by men and women to research health information increased over time. Facebook use declined over time (*P*<.001), whereas use of YouTube (*P*=.03) and Instagram (*P*<.001) increased, especially among women and more US acculturated youths.

**Conclusion:**

Social media is ubiquitous in Latino adolescents’ lives and may be a powerful mode for public health intervention delivery.

## Introduction

### Background

Today, adolescents are consumed by a dynamic, technology-filled world. Social media, such as social networking sites (SNSs, ie, platforms utilizing the Internet and mobile technology to enable social interactions) [1] and short message services (SMSs or texting) allow adolescents to connect to peer and other social networks that are larger and more diverse than they would normally have access to face-to-face. Adolescents are the most extensive users of new technology and are more likely to be connected to the virtual world, regardless of socioeconomic status, family structure, or race [2]. In fact, many adolescents use social media to interact with peers and others whom they may not even know on a personal basis [3]. Given that almost all US youths use social media in their daily lives, research suggests these media may have negative consequences related to alcohol use, sex, suicide ideation, and bullying [4-7]. At the same time, the rise in social media use over the last decade has spawned an increase in using media platforms to deliver public health messages and information [8-11]. Many health interventions target adolescents and young adults because of their increased risk behavior during this time and their extensive social media use. However, little is known about how, why, and how much ethnic and minority youths use social media, and if social media are an avenue to reach specific populations within minority communities for health interventions.

Latino youths are one of the fastest-growing minority youth populations in the United States, and by 2025, it is estimated that they will comprise one quarter of the total US youth population [12]. Despite being a minority population, typically characterized as a “low-income” group, a recent Pew Internet and American Life Project report noted that 86% of Latino youths own a cell phone, similar to whites (84%) and blacks (90%) [13]. Furthermore, Latinos are just as likely as their white and black peers to own a smartphone—49% versus 46% and 50%, respectively. Latino youths are also extensive users of mobile technology, particularly SMS and social networking, with 55% of Latino youths using SMS as their primary method to communicate, and sending a median of 100 SMS/day [2,14].

Previous studies have indicated that adolescent women and men have very different communication styles. Women use communication to develop more intimate social relationships, whereas men tend to restrict emotional expressions [15,16]. Thus, it is plausible that adolescent women and men communicate differently through social media as well. Traditionally, men watch more television and share videos online, whereas women blog, email, or instant message [17]. In a national study, female adolescents (84%) were more likely than male adolescents (79%) to have a social networking account, and they sent/received a median of 100 SMS/day, compared with 50 SMS/day that men sent/received [2]. Different communication styles may also exist when using mobile technology for obtaining health information or initiating health discussions. Examining whether this phenomenon exists in the Latino community is particularly important because there is a strong emphasis on distinct sex differences between men and women. Sex is viewed as an organizing feature of family life during the socialization process [18], and Latino parents are generally considered as being more protective and stricter about their daughters’ activities as compared with their sons’ activities [19]. Thus, understanding sex differences is crucial to the development of mobile health interventions, as this may play a key role in understanding who is more likely to participate in these types of interventions.

Acculturation, defined as a cultural modification by adapting to or borrowing traits from another culture [20], is another factor that may influence social media utilization and behavior. Previous studies have found a strong relationship between acculturation and risk behaviors among Latino youths [21,22]. Preliminary evidence showing differences in utilization of social media by acculturation was reported in a descriptive study [14]. The study [14] examined proxy measures of acculturation, nativity, and language spoken at home, and reported that 65% of US native teens communicate with friends using SMS versus 26% of foreign-born teens. Other findings from the same study showed that 68% of English-dominant and 50% of bilingual young Latinos used SMS daily, compared with only 19% of Spanish-dominant Latinos. Furthermore, native-born Latinos were 3 times more likely than foreign-born youths to use SNSs to socialize with friends [14].

### Study Objective

The purpose of this study was to examine Latino adolescents’ social media utilization and behavior over a 16-month period, and to assess whether changes in use differed by sex and acculturation.

## Methods

### Participants

The data for this study were derived from self-identifying Latino adolescents aged 13-19 (mean 15.33, SD 1.03), recruited from 12 public high schools in Maryland. Participants completed baseline and 16-month follow-up surveys conducted as part of a program evaluation of the Empowering Latino Youth Project (ELYP) between spring 2012 and fall 2013 (n=555). ELYP is a 5-year cluster-randomized control trial of a teen pregnancy prevention program. Parental consent and youths’ assent to participate in ELYP were obtained. Because the data are from an intervention study, all final analyses controlled for the intervention/control group. The control group was an attention-control program that focused on fitness and nutrition.

### Data Collection

To ensure privacy and reduce reporting bias, surveys were administered via individual laptops with audio capability for youths with low-literacy levels. Study participants chose to complete the survey in English or Spanish and were given US$ 10 gift cards for completing the baseline survey and US$ 20 gift cards for completing the 16-month survey. The survey instruments were translated and back-translated, and pretested for readability and accuracy. Upon survey completion, the data were stored in an encrypted file to be read only by the survey design software *Snap Surveys* [23].

### Measures

Demographic and background variables included age, sex, grade, US born, years in the United States, and acculturation. Age was calculated as a continuous variable from the participants’ self-reported date of birth. Participants self-reported the variables “US born” or “years they have been in the United States,” which was categorized into US born, 0-3 years, 4-10 years, and 10+ years.

###  Acculturation

This study used an adapted version of a validated bilinear scale consisting of items from the US and Latino cultural identity subscales of the Abbreviated Multidimensional Acculturation Scale [24]. Participants were asked to indicate their level of agreement with 6 statements measured on a 5-point Likert-type scale (1=strongly disagree, 5=strongly agree): “I am proud of being (Latino/American),” “I feel good about being (Latino/American),” and “I think of myself as being (Latino/American).” The 2 subscales, measuring US culture and Latino culture had very high internal consistency (Cronbach alpha=.9298 and .9517, respectively). After creating the subscales, we conducted a 4-group k-means cluster analysis. Each cluster contains participants such that the degree of association is strong between members of the same cluster and weak between members of different clusters. Each cluster describes the category to which participants belong, including high Latino-high American, low Latino-high American, high Latino-low American, and low Latino-low American identities.

###  Social Media Use

Social media use includes SMS, Internet, and social media questions adapted from the Pew Internet Project’s 2011 teen survey [2]. Participants reported if they had a cell phone, used SMS, and the frequency of SMS/day (high SMS > 100/day; low SMS ≤ 100/day). SMS frequency was dichotomized based on Pew data that suggested that the median number of SMS/day for Hispanic adolescents is 100 [13]. Participants reported how often they texted their friends, parents, and a boy/girlfriend (1=less often or never, 4=several times a day), which was dichotomized (at least once/day versus less often). In addition, participants with a cell phone reported the following behaviors using their phone: send or receive email, take pictures, play music, send or receive instant messages, record videos, play games, or access Internet.

Participants were asked if they use the Internet; if so, for what purposes and how often (0=never, 6=several times a day). Finally, participants were asked if they had accounts on SNS: Facebook, Myspace, Twitter, Yahoo!, YouTube, Instagram, Tumblr, Google Buzz, Flickr, and Ustream. Those with any of these accounts reported certain behaviors (ie, instant messaging, posting comments, private messaging, tagging people, and posting updates or videos) and frequency of logging that was dichotomized into daily login versus less frequent.

### Statistical Analysis

Analyses were conducted based on participants who completed both the baseline (T1) and 16-month follow-up surveys (T2). Bivariate analyses were conducted to examine the proportions of social media use at different time points by sex and acculturation. To adjust for correlation among repeated measures within individuals, we examined the prevalence of social media use and frequency at T1 and T2 using generalized estimating equations (GEEs) with an unstructured correlation structure and robust standard errors to calculate parameter coefficients and 95% CI. Final GEE models measured whether social media behaviors and frequencies between T1 and T2 differed by sex or acculturation, after controlling for age and the intervention/control group. All analyses were conducted in STATA 12.0 (StataCorp, College Station, TX, USA) [25]. This study was reviewed and approved by the George Washington University Internal Review Board (IRB No 011217).

## Results

### Demographics of the Study Sample

Table 1 lists the self-reported demographic characteristics of the study sample at baseline. There were slightly more female (325, 58.6%) than male participants (230, 41.4%). The majority of participants were in ninth grade (404, 72.8%) and slightly less than half were born in the United States (268, 48.3%). Of those born outside of the United States, 24.5% (136) had been in the United States for 0-3 years, 18.4% (102) for 4-10 years, and slightly less than 5% (27) for 10 years or more. As much as 50.1% (278) of participants reported high Latino and high American cultural identities, whereas 3% (17) reported low cultural identity on both scales.

**Table 1:** Study sample characteristics at baseline (N=555)^a^.

Variables		Category	n (%)
**Sex**			
		Men	230 (41.4)
		Women	325 (58.6)
**Grade**			
		9th	404 (72.8)
		10th	151 (27.2)
**Length of time in the United States**			
		US born	268 (48.3)
		0-3 years	136 (24.5)
		4-10 years	102 (18.4)
		10+ years	27 (4.9)
		Missing	22 (4.0)
**Acculturation scores**			
	Latino culture^b^		0.88 (4.6)^c^
	**American culture** ^d^		1.12 (4.0)^c^
		High Latino High American	278 (50.1)
		Low Latino High American	121 (21.8)
		High Latino Low American	112 (20.2)
		Low Latino Low American	17 (3.1)
		Missing	27 (4.9)

^a^The data for this study were derived from self-identifying Latino adolescents aged 13-19 (mean 15.33, SD 1.03).

^b^Cronbach alpha=.9517

^c^Data presented as mean (SD)

^d^Cronbach alpha=.9298

In terms of social media use and behaviors (Table 2), a vast majority (488, 87.9%) owned or had access to a cell phone at baseline, and by the 16-month follow-up (T2), nearly all participants (494, 89.0%) gained access to a cell phone. Of the 7 measured activities on cell phones, emailing (334/466, 71.7%, versus 427/485, 88.0%, *P*<.001) and accessing Internet (430/479, 89.8%, versus 474/488, 97.1%, *P*<.001) had the largest percentage point increase over the 16 months. Ninety-five percent (467/488) of participants used SMS at baseline and nearly all (486/493, 98.6%) used SMS at follow-up (*P*=.01).

**Table 2:** Social media use and behaviors at baseline (T1) and 16-month follow-up (T2).

Variables of interest		T1, n/N (%)	T2, n/N (%)	*P* value
Cell phone access (yes)		488/555 (87.9)	494/555 (89.0)	.04
Missing data on cell phone access		5/555 (0.9)	18/555 (3.2)	—
**Cell phone activities**				
	Email	334/466 (71.7)	427/485 (88.0)	<.001
	Pictures	451/476 (94.8)	477/489 (97.6)	.01
	Listen to music	445/482 (92.3)	475/492 (96.5)	.001
	Instant messages	438/474 (92.4)	469/489 (95.9)	.01
	Record videos	381/467 (81.6)	427/478 (89.3)	<.001
	Play games	394/470 (83.8)	412/478 (86.2)	—
	Access Internet	430/479 (89.8)	474/488 (97.1)	<.001
Mean number of activities on cell phone (SD)		5.9 (1.61)	6.3 (1.26)	<.001
SMS use		467/488 (95.7)	486/493 (98.6)	.01
More than 100 SMS/day		149/438 (34.0)	135/484 (27.9)	.02
100 or fewer SMS/day		289/438 (66.0)	349/484 (72.1)	—
SMS parents at least once/day		239/448 (53.4)	260/481 (54.1)	—
SMS friends at least once/day		403/460 (87.6)	418/486 (86.0)	—
SMS boy/girlfriend at least once/day		283/437 (64.8)	322/469 (68.7)	—
Internet use		533/550 (96.9)	529/537 (98.5)	—
**Internet activities**				
	Send/read email	420/516 (81.4)	464/523 (88.7)	<.001
	Research health information	184/504 (36.5)	307/518 (59.3)	<.001
	Information for homework or school	454/517 (87.8)	486/521 (93.3)	.001
Use Internet once/day		415/531 (78.2)	416/529 (78.6)	—
**Has social networking account**		528/552 (95.7)	518/534 (97.0)	—
	Facebook	458/552 (83.0)	390/534 (73.0)	<.001
	Twitter	324/552 (58.7)	298/534 (55.8)	—
	YouTube	341/552 (61.8)	357/534 (66.9)	.03
	Instagram	82/555 (14.8)	130/555 (23.4)	<.001
**Social networking activities**				
	Send instant messages	421/512 (82.2)	412/507 (81.3)	—
	Post comments	440/522 (84.4)	425/520 (81.7)	—
	Send private messages	357/519 (68.8)	353/509 (69.4)	—
	Tag people	387/512 (75.6)	385/510 (75.5)	—
	Post status updates	391/508 (77.0)	354/505 (70.1)	.003
	Post photos or videos	466/517 (90.1)	454/512 (88.7)	—
Logging in to social networking sites ≥1 time/day		417/527 (79.1)	426/519 (82.1)	—

At both baseline and follow-up survey points, Internet use was above 95% (533/550 and 529/537, respectively). Utilization of the Internet to research health information had the largest gain (184/504, 36.5%, versus 307/518, 59.3%, *P*<.001). Social networking remained high between survey points with over 93% (528/552 and 518/534, respectively) having at least one SNS account. Facebook use significantly declined between the 2 surveys (458/552, 83.0%, versus 390/534, 73.0%, *P*<.001), whereas use of YouTube (341/552, 61.8%, versus 357/534, 66.9%, *P*=.03) and Instagram (82/555, 15%, versus 130/555, 23.4%, *P*<.001) significantly increased.

### Sex

Results indicate that women were significantly more likely to use SMS at baseline (see Multimedia Appendix 1), but men were more likely to SMS a girl/boyfriend (124/175, 70.8%, versus 159/262, 60.6%, *P*=.03). Women were more likely than men to use the Internet for schoolwork (277/302, 91.7%, versus 177/215, 82.3%, *P*=.001) and to have a Twitter or Instagram account. Women were more active on SNS with tagging people and posting status updates or photos/videos. Women also had slightly more SNS accounts at baseline as compared with men (mean 5.18 versus 4.92, *P*=.048).

At the 16-month follow-up (T2), women reported higher cell phone access than men (*P*=.005), and were more likely to SMS their parents (*P*=.003). There was an increase in using the Internet for health information by T2 for both men and women, but only significant for women (202/314, 64.3%, versus 105/204, 51.4%, *P*=.004). At T2, women continued to have a significantly higher presence on Twitter and Instagram (tagging people, posting status updates, and posting photos/videos). Women were also significantly more likely to login to an SNS at least once a day at T2 (265/311, 85.2%, versus 161/208, 77.4%, *P*=.02).

### Acculturation

At baseline, participants in the *high Latino-high American* category used their cell phone for significantly more activities compared with participants in the *high Latino-low American* category (mean 6.14 versus 5.57, *P*=.02). Sending SMS to friends also significantly varied between cultural identities with 91.0% (213/234) of those in the *high Latino-high American* category sending more SMS to friends, compared with 83% (88/105) in the *low Latino-high American* category.

At the 16-month follow-up (T2), there was an increase in differences between acculturation categories. Most notably, those in the *high Latino-high American* category performed significantly more activities with their cell phone than those in the *low Latino-high American* and the *high Latino-low American* categories (mean 6.53 versus 6.04 and 5.92, respectively, *P*=.009). Participants in the *high Latino-high American* and *low Latino-high American* categories were significantly less likely to have a Facebook account compared with the *high Latino-low American* and the *low Latino-low American* categories (194/285, 68.0%, and 88/122, 72%, versus 82/99, 83%, and 16/18, 89%, respectively, *P*=.01). By contrast, and compared with other categories, the *high Latino-high American* category was significantly more likely to have a Twitter (181/285, 63.5%) or Instagram (79/287, 28%) account (*P*<.001 and *P*=.04, respectively).

### Multivariate GEE Models

Final multivariate GEE models included change between baseline and 16-month follow-up for selected social media variables, stratified by sex and acculturation categories and controlling for age and the intervention/control group (Table 3). Over time, access to a cell phone (adjusted odds ratio, aOR, 2.34, 95% CI 1.126-4.844) and Internet use (aOR 10.40, 95% CI 1.829-59.194) significantly increased for women. Women were more likely to research health information over time (aOR 2.29, 95% CI 1.576-3.341), and although both men and women experienced declines in Facebook use, women had a larger decline (aOR 0.260, 95% CI 0.167-0.406 versus aOR 0.318, 95% CI 0.169-0.600). Between baseline and T2, both men and women increased the mean number of activities performed on a cell phone (adjusted beta coefficient men=.551, 95% CI 0.222-0.879 and adjusted beta coefficient women=.675, 95% CI 0.413-0.938), as well as use of YouTube and Instagram (Table 3).

The *high Latino-high American* cultural group reported a significant increase in the number of activities performed on a cell phone over time (adjusted beta coefficient .517, 95% CI 0.262-0.772), whereas the *low Latino-high American* group significantly declined in high-frequency SMS (aOR 0.484, 95% CI 0.238-0.983). Except the *low Latino-low American* group, all acculturation group types significantly increased in researching health information on the Internet with the low Latino-high American group experiencing the greatest increase (aOR 2.54, 95% CI 1.398-4.606). Excluding the *low Latino-high American* group, all acculturation groups significantly decreased Facebook use over time. YouTube use increased for both high Latino groups, and Instagram use increased for all groups except the *low Latino-low American* group, which was omitted because there were no Instagram users in that group.

**Table 3:** Changes in social media access and behaviors between baseline (T1) and 16-month (T2) follow-up, stratified by sex and acculturation categories^a^.

Social media variables	Sex		Acculturation categories			
	Men, Beta or OR (95% CI)^b^	Women, Beta or OR (95% CI)^b^	High Latino-high American, Beta or OR (95% CI)^b^	Low Latino-High American, Beta or OR (95% CI)^b^	High Latino-low American, Beta or OR (95% CI)^b^	Low Latino-low American, Beta or OR (95% CI)^b^
Cell phone access (yes)	0.804 (0.452-1.429)	2.34 (1.126-4.844)^c^	1.67 (0.842-3.318)	0.453 (0.136-1.504)	1.38 (0.603-3.172)	Omitted^d^
Mean number of activities on cell phone^e^	.551 (0.222-0.879)^f^	.675 (0.413-0.938)^f^	.517 (0.262-0.772)^f^	.458 (-0.016 to 9.32)	.275 (-0.224 to 0.773)	.571 (-0.191 to 1.333)
Send/receive more than 100 SMS/day	0.938 (0.571-1.541)	0.844 (0.568-1.254)	0.956 (0.630-1.451)	0.484 (0.238-0.983)^g^	1.45 (0.651-3.215)	1.22 (0.182-8.227)
Internet use	1.76 (0.343-9.043)	10.40 (1.829-59.194)^c^	1.01 (0.986-1.043)	1.03 (0.976-1.085)	1.07 (0.995-1.144)	0.959 (0.884-1.041)
Research health information on Internet	1.40 (0.924-2.135)	2.29 (1.576-3.341)^f^	1.573 (1.041-2.378)^g^	2.54 (1.398-4.606)^c^	1.97 (1.019-3.809)^g^	1.86 (0.382-9.072)
Facebook	0.318 (0.169-0.600)^f^	0.260 (0.167-0.406)^f^	0.173 (0.100-0.300)^f^	0.541 (0.273-1.070)	0.381 (0.148-0.984)^g^	0.001 (0.004-0.232)^g^
Twitter	1.49 (1.056-2.107)^g^	0.965 (0.690-1.351)	1.06 (0.739-1.529)	0.867 (0.504-1.493)	1.03 (0.603-1.758)	0.505 (0.059-4.295)
YouTube	1.89 (1.228-2.916)^f^	1.87 (1.327-2.637)^f^	1.86 (1.247-2.786)^c^	1.29 (0.734-2.278)	2.68 (1.445-4.968)^c^	0.350 (0.061-2.003)
Instagram	3.99 (1.854-8.571)^f^	3.22 (2.095-4.950)^f^	1.19 (1.101-1.290)^f^	1.25 (1.139-1.374)^f^	1.16 (1.049-1.292)^c^	Omitted^d^

^a^All models controlled for age and the intervention/control group.

^b^Odds ratios (OR) or beta coefficients represent the change in social media variables from baseline to 16-month follow-up stratified by sex or acculturation categories.

^c^ *P*<.01

^d^Omitted odds ratios are due to perfect prediction of the outcome variable in that group.

^e^Data presented as beta coefficient (95% CI)

^f^ *P*<.001

^g^ *P*<.05

## Discussion

### Principal Findings

To our knowledge, this is the first longitudinal study to examine specific social media use and behaviors among Latino youths by sex and acculturation. Consistent with prior research on overall social media use [2,14], participants in this sample were extensive social media users, but differences by sex and acculturation did emerge. This is important for public health practitioners who target minority youths as part of mobile health interventions.

### Social Media Use

As expected, social media-related access, utilization, and activities increased over the 16 months. Using cell phones increasingly for emailing and Internet activities at follow-up were likely a result of maturity, with email being more professional and used for schoolwork. A particularly interesting finding was the large increase in searching for health information online between the 2 periods. This is consistent with prior studies of youths reporting that they would rather receive information online (versus traditional forms), especially sexual health information [26-29]. The participants of this study belonged to the 9th to 10th grade, which corresponds to a period when adolescents are becoming more autonomous, curious, and searching for their own information as opposed to asking a parent or guardian, or even a health care provider [26]. Future health-focused interventions should consider how youths could receive accurate and developmentally salient information via the Internet and mobile technologies.

### Sex Differences

Women were earlier adopters of newer technology and applications such as Instagram, and were more active on SNSs. This is consistent with prior research suggesting that the primary purpose for female communication is to build connections and acquire confirmation and support [16]. One possible explanation for this finding is that adolescent women may use social media as a way to develop intimacy and share their feelings with their peers and social networks [30]. However, being more expressive on social media can also lead to more vulnerability and potential cyber bullying or cruelty on these platforms [31,32], and therefore, it is crucial to train youths on how to safely use these new and changing platforms. Women were more active on SNSs, but they also used the Internet for positive purposes, such as researching health-related information.

Despite Latino parents generally being more protective of women [18], results suggest that women in this sample obtained cell phone access at a greater rate than men. One plausible explanation is that parents may relax their protective principles when it comes to cell phone use compared with in-person exposure and relationships because they feel they can monitor their children more closely with a cell phone [33,34]. Alternatively, participants come from a large immigrant population where parents may not be aware of how much time is spent on media or their children’s behaviors on these platforms. Immigrant parents might have less strict principles regarding limiting excessive use and monitoring mobile activities or networks. Nonetheless, this provides an opportunity for mobile health interventions to reach Latina women and provide targeted health information—particularly in protective environments where it is taboo to discuss reproductive health issues [35].

### Acculturation Differences

Similar to previous research, participants belonging to the high Latino and American cultural group were more active on cell phones over time. Prior research suggests that higher acculturation levels may lead to increased risk behaviors [14,21]. At the same time, individuals in this group can be easily targeted with mobile-based interventions based on their social media access and use.

The low Latino-high American group was the only group to show a decline in high-frequency SMS. One explanation for this finding is that the higher American groups may be faster to adopt newer platforms and apps compared with the low American identity groups. Traditional SMS may also be declining because of new features available in popular apps, such as commenting on photos in Instagram and other interactive communication features. However, the insignificant change in the high Latino-high American group could be explained by SMS being a low-cost way to communicate with people across the world, especially for this sample of immigrant Latinos where family members may remain in home countries. Although both groups self-identify with higher American culture, the high Latino group may still have strong family ties in their home country, and thus, SMS remains an important communication tool.

### Limitations

Despite the longitudinal nature of this study, results should be considered in light of limitations. First, the data were self-reported and could be subject to response bias due to social desirability. However, this was attenuated by the use of personal laptops and audio capability to increase data dependability [36]. Second, because several social media outcomes were examined, there is a possibility of a multiple comparisons problem. However, an increased sample size is a method of limiting multiple comparison problems [37], and the sample size of 555 in this study was deemed sufficient to detect differences between groups and not due to random chance alone.

### Conclusions

We know young people from diverse cultures and backgrounds quickly adopt social media, and it evolves rapidly. The public health community has a unique opportunity to disseminate health-related messages via digital platforms for high-risk Latino youths, given their rapid adoption of newer applications and sites, and this is especially important for Latino youths who have been cited as harder to reach or retain in health-promotion programs [38,39]. At the same time, policies and programs must formulate better methods for youths to safely engage with social media [7,8,40,41].

## Abbreviations

aOR: adjusted odds ratio
ELYP: Empowering Latino Youth Project
GEEs: generalized estimating equations
SMS: short message service
SNSs: social networking sites

## Appendix

Multimedia Appendix 1 Bivariate relationships of social media use at T1 and T2, by sex and acculturation.
