# Supplementary material for: Evaluation of Social Media Utilization by Latino Adolescents: Implications for Mobile Health Interventions
Source: JMIR Mhealth Uhealth. 2015 Sep 29;3(3):e89. doi: 10.2196/mhealth.4374 (PMC4704907; doi:10.2196/mhealth.4374)
Supplement: Multimedia Appendix 1 [file mhealth_v3i3e89_app1.pdf]

Variables		Sex						Acculturation									
		T1			T2			T1					T2				
		Men, n/N (%)	Women, n/N (%)	*P* value	Men, n/N (%)	Women, n/N (%)	*P* value	High Latino-high American, n/N (%)	Low Latino-high American, n/N (%)	High Latino-low American, n/N (%)	Low Latino-low American, n/N (%)	*P* value	High Latino-high American, n/N (%)	Low Latino-high American, n/N (%)	High Latino-low American, n/N (%)	Low Latino-low American, n/N (%)	*P* value
Cell phone access (yes)		202/226 (89.4)	286/324 (88.3)	.69	190/216 (88.0)	304/321 (94.7)	.005	245/275 (89.1)	110/120 (91.7)	93/111 (83.8)	15/17 (88.2)	.29	267/287 (93.0)	113/123 (91.9)	86/99 (86.9)	18/18 (100.0)	.14
**Cell phone activities**																	
	Email	141/190 (74.2)	193/276 (69.9)	.31	163/186 (87.6)	264/299 (88.3)	.83	182/243 (74.9)	75/105 (71.4)	56/87 (64.4)	10/12 (83.3)	.23	240/264 (90.9)	97/111 (87.4)	69/84 (82.1)	14/17 (82.4)	.14
	Pictures	180/194 (92.8)	271/282 (96.1)	.11	181/187 (96.8)	296/302 (98.0)	.4	234/243 (96.3)	101/109 (92.7)	85/91 (93.4)	14/14 (100.0)	.35	261/265 (98.5)	105/119 (95.5)	83/86 (96.5)	18/18 (100.0)	.29
	Listen to music	187/200 (93.5)	258/282 (91.5)	.41	184/190 (96.8)	291/302 (96.4)	.78	224/243 (92.2)	101/110 (91.8)	84/92 (91.3)	14/15 (93.3)	.99	261/267 (97.8)	107/112 (95.5)	79/85 (92.9)	18/18 (100.0)	.15
	Instant Messages	176/194 (90.7)	262/280 (93.6)	.25	183/188 (97.3)	286/301 (95.0)	.21	231/243 (95.1)	98/109 (89.9)	79/88 (89.8)	14/14 (100.0)	.13	259/267 (97.0)	106/111 (95.5)	79/85 (92.9)	18/18 (100.0)	.3
	Record Videos	151/191 (79.1)	230/276 (83.3)	.24	159/184 (86.4)	268/294 (91.2)	.1	205/242 (84.7)	88/106 (83.0)	63/86 (73.3)	12/15 (80.0)	.12	243/263 (92.4)	89/109 (81.7)	72/81 (88.9)	16/16 (94.1)	.02

	Play games	163/195 (83.6)	231/275 (84.0)	.91	162/186 (87.1)	250/292 (85.6)	.65	209/240 (87.1)	87/108 (80.6)	71/89 (79.8)	12/14 (85.7)	.28	236/263 (89.7)	90/109 (82.6)	65/81 (80.3)	16/18 (88.9)	.09
	Access Internet	178/196 (90.8)	252283 (89.1)	.53	183/188 (97.3)	291/300 (97.0)	.83	220/243 (90.5)	98/110 (89.1)	80/91 (87.9)	14/15 (93.3)	.86	259/265 (97.7)	107/111 (96.4)	81/85 (95.3)	18/18 (100.0)	.56
Mean number of activities on cell phone (SD)		5.8 (0.12)	6.0 (0.09)	.19	6.3 (0.10)	6.3 (0.09)	.45	6.1 (1.45)[a]	5.9 (1.73)	5.6 (1.60)[a]	6.0 (1.20)	.02	6.5 (1.07)[a]	6.0 (1.43)[a]	5.9 (1.51)[a]	6.46 (0.66)	.009
SMS use		188/202 (93.1)	279/286 (97.6)	.02	188/190 (99.0)	298/303 (98.4)	.59	236/245 (96.3)	105/110 (95.5)	91/93 (97.9)	15/15 (100.0)	.7	264/267 (98.9)	111/113 (98.2)	83/85 (97.7)	18/18 (100.0)	.79
Send/receive more than 100 SMS/day		53/178 (29.8)	96/260 (36.9)	.12	45/188 (23.9)	90/296 (30.4)	.12	77/224 (34.4)	40/101 (39.6)	21/83 (25.3)	6/14 (42.9)	.2	83/264 (31.4)	22/111 (19.8)	23/81 (28.4)	4/18 (22.2)	.14
SMS parents at least once/day		84/175 (48.0)	155/273 (56.8)	.07	84/185 (45.4)	176/296 (59.5)	.003	129/233 (55.4)	50/101 (49.5)	44/87 (50.6)	5/13 (38.5)	.52	147/261 (56.3)	56/110 (50.9)	38/83 (45.8)	11/17 (64.7)	.26
SMS friends at least once/day		156/182 (85.7)	247/278 (88.9)	.32	157/188 (83.5)	261/298 (87.6)	.21	213/234 (91.0)[a]	88/105 (83.8)[a]	77/90 (85.6)	9/13 (69.2)	.04	225/264 (85.2)	89/111 (80.2)	77/83 (92.8)	17/18 (94.4)	.06
SMS boy/girlfriend at least once/day		124/175 (70.9)	159/262 (60.7)	.03	135/183 (73.8)	187/286 (65.4)	.056	139/227 (61.2)	65/99 (65.7)	57/85 (67.1)	12/14 (85.7)	.25	173/257 (67.3)	65/106 (61.3)	63/81 (77.8)	14/17 (82.4)	.06
Internet use		221/225 (98.2)	312/325 (96.0)	.14	210/216 (97.2)	319/321 (99.4)	.04	272/276 (98.6)	119/121 (98.4)	105/111 (94.6)	16/16 (100.0)	.1	284/287 (99.0)	122/123 (99.2)	96/99 (97.0)	17/18 (94.4)	.23

**Internet activities**																	
	Send/read email	169/217 (77.9)	251/299 (83.9)	.08	173/206 (84.0)	291/317 (91.8)	.006	227/269 (84.4)	91/113 (80.5)	75/99 (75.8)	12/15 (80.0)	.29	259/283 (91.5)	102/121 (84.3)	81/92 (88.0)	13/17 (76.5)	.07
	Research health information	77/209 (36.8)	107/295 (36.3)	.9	105/204 (51.5)	202/314 (64.3)	.004	93/267 (34.8)	44/109 (40.4)	33/96 (34.4)	5/15 (33.3)	.75	159/280 (56.8)	76/117 (65.0)	54/94 (57.5)	10/17 (58.8)	.5
	Information for homework or school	177/215 (82.3)	277/302 (91.7)	.001	188/207 (90.8)	298/314 (94.9)	.07	241/270 (89.3)	95/112 (84.8)	89/101 (88.1)	12/14 (85.7)	.67	264/281 (94.0)	109/119 (91.6)	88/94 (93.6)	16/17 (94.1)	.86
Using Internet once/day		171/221 (77.4)	244/310 (78.7)	.71	165/210 (78.6)	251 (78.7)	.98	220/271 (81.2)	92/119 (77.3)	75/104 (72.1)	14/16 (87.5)	.22	228/284 (80.3)	95/122 (77.9)	72/96 (75.0)	13/17 (76.5)	.54
**Social networking sites**																	
	Facebook	188/229 (82.10)	270/323 (83.6)	.65	165/213 (77.5)	225/321 (70.1)	.06	226/277 (81.6)	95/120 (79.2)	99/111 (89.2)	15/17 (88.2)	.18	194/285 (68.1)[a]	88/122 (72.1)[a]	82/99 (82.8)[a]	16/18 (88.9)	.01
	Twitter	106/229 (46.3)	218/323 (67.5)	<.001	100/213 (47.0)	198/321 (61.7)	.001	180/277 (65.0)	71/120 (59.2)	58/111 (52.3)	8/17 (47.1)	.08	181/285 (63.5)[a]	60/122 (49.2)[a]	49/99 (49.5)	3/18 (16.7)[a]	<.001
	YouTube	151/229 (65.9)	190/323 (58.8)	.09	149/213 (70.0)	208/321 (64.8)	.22	184/277 (66.4)[a]	77/120 (64.2)[a]	57/111 (51.4)[a]	14/17 (82.4)[a]	.01	197/285 (69.1)	77/122 (63.1)	65/99 (65.7)	12/18 (66.7)	.68
	Instagram	16/230 (6.9)	66/325 (20.3)	<.001	27/225 (15.8)	101/330 (30.6)	<.001	52/278 (18.7)	13/121 (10.7)	16/112 (14.3)	0/17 (0.0)	.05	79/287 (27.5)[a]	29/123 (23.6)	21/99 (122)[a]	0/18 (0.0)	.04
**Social networki**																	

**ng activities**																	
	Send instant messages	172/202 (85.2)	249/310 (80.3)	.16	164/203 (80.8)	248/304 (81.6)	.82	227/265 (85.7)	88/111 (79.3)	82/102 (80.4)	13/17 (76.5)	.34	223/276 (80.8)	96/111 (86.5)	73/95 (76.8)	13/17 (76.5)	.32
	Post comments	172/207 (83.1)	268/315 (85.1)	.54	162/209 (77.5)	263/311 (84.6)	.04	231/267 (86.5)	93/112 (83.0)	89/107 (83.2)	13/16 (81.3)	.74	240/279 (86.0)	83/116 (71.6)	78/98 (79.6)	15/17 (88.2)	.007
	Send private messages	145/206 (70.4)	212/313 (67.7)	.52	139/205 (67.8)	214/304 (70.4)	.53	189/267 (70.8)	69/111 (62.2)	74/105 (70.5)	14/16 (87.5)	.14	192/276 (69.6)	82/114 (71.9)	63/93 (67.7)	12/17 (70.6)	.93
	Tag people	137/203 (67.5)	250/309 (80.9)	.001	141/204 (69.1)	244/306 (79.7)	.006	216/267 (80.9)	83/110 (75.5)	70/101 (69.3)	10/16 (62.5)	.05	221/278 (79.5)	77/112 (68.8)	70/95 (73.7)	11/17 (64.7)	.09
	Post status updates	144/200 (72.0)	247/308 (80.2)	.03	132/204 (64.7)	222/301 (73.8)	.03	208/266 (78.2)	79/107 (73.8)	78/101 (77.2)	12/16 (75.0)	.84	188/273 (68.9)	80/113 (70.0)	64/94 (68.1)	15/17 (88.2)	.39
	Post photos or videos	175/204 (85.8)	291/313 (93.0)	.007	171/206 (83.0)	283/306 (92.5)	.001	250/268 (93.3)	97/110 (88.2)	92/104 (88.5)	13/16 (81.3)	.15	252/277 (91.0)	94/113 (83.2)	85/96 (88.5)	15/16 (93.8)	.15
Mean number of activities on social networking site (SD)		4.9 (0.13)	5.2 (0.09)	.048	4.9 (0.15)	5.1 (0.11)	.2	5.3 (1.56)	5.0 (1.86)	4.9 (1.81)	4.9 (2.38)	.13	5.2 (1.57)	5.0 (1.78)	4.6 (1.85)	5.1 (2.06)	.1
Logging in to social networking sites one or more times/day		160/211 (75.8)	257/316 (81.3)	.13	161/208 (77.4)	265/311 (85.2)	.02	222/168 (82.8)	85/112 (75.9)	82/106 (77.4)	13/17 (76.5)	.38	236/280 (84.3)	90/115 (78.3)	79/97 (81.4)	12/17 (70.6)	.31

[a]Categories that are significantly different from each other.
